# Impact of Active learning model and prior knowledge on discovery time of elusive relevant papers: a simulation study

**DOI:** 10.1186/s13643-024-02587-0

**Published:** 2024-07-08

**Authors:** Fionn Byrne, Laura Hofstee, Jelle Teijema, Jonathan De Bruin, Rens van de Schoot

**Affiliations:** 1 Department of Information and Computing Science, Faculty of Science, Utrecht University, Utrecht, The Netherlands; 2 Department of Methodology and Statistics, Faculty of Social and Behavioral Sciences, Utrecht University, Utrecht, The Netherlands; 3 Research and Data Management Services, Utrecht University, Utrecht, The Netherlands

**Keywords:** Time to discovery, Active learning, Systematic reviews, Screening tools

## Abstract

Software that employs screening prioritization through active learning (AL) has accelerated the screening process significantly by ranking an unordered set of records by their predicted relevance. However, failing to find a relevant paper might alter the findings of a systematic review, highlighting the importance of identifying elusive papers. The time to discovery (TD) measures how many records are needed to be screened to find a relevant paper, making it a helpful tool for detecting such papers. The main aim of this project was to investigate how the choice of the model and prior knowledge influence the TD values of the hard-to-find relevant papers and their rank orders. A simulation study was conducted, mimicking the screening process on a dataset containing titles, abstracts, and labels used for an already published systematic review. The results demonstrated that AL model choice, and mostly the choice of the feature extractor but not the choice of prior knowledge, significantly influenced the TD values and the rank order of the elusive relevant papers. Future research should examine the characteristics of elusive relevant papers to discover *why* they might take a long time to be found.

## Introduction

Systematic reviews play a crucial role in synthesizing research findings to address specific scientific questions. One of the persistent challenges in this process is the substantial time required to screen and evaluate the relevance of the literature. Historically, this issue has been noted by studies such as Bastian et al. [3], and Borah et al. [5], highlighting its long-standing nature. Screening prioritization through active learning (AL) has enabled the screening process to be sped up significantly by ranking an unordered set of records by their predicted relevance [17, 23, 25, 29, 32]. It enables the user to theoretically find relevant papers by screening only a fraction of the most likely relevant records [9].

With AL, just like with a classical systematic review pipeline, the process starts with a pool of unlabeled records with meta-data containing titles and abstracts of scientific papers retrieved from a search. This is followed by constructing a *training set* consisting of at least one labeled relevant and irrelevant record provided by the annotator. Next, a model needs to be selected, including a *feature extraction* technique (which translates text into values that a machine can process) and a *classification algorithm* (i.e., a machine learning model that produces relevance scores). The record with the highest relevance score is shown to the annotator [13]. The annotator screens this record and provides a label: relevant or irrelevant, and it goes back to the training set. This cycle is repeated until the annotator has seen all relevant records, with the goal of saving time by screening fewer records than exist in the entire pool.

Research demonstrates that AL can significantly reduce the workload involved in screening records, as evidenced by studies that report marked time savings [18, 24]. Moreover, AL facilitates the inclusion of a broader array of papers during the search phase, a benefit detailed in the work of [6], where a team screened over 11,000 most likely relevant papers out of a set of 165.046 hits to find out what factors and interaction of factors contribute to the onset, maintenance, and relapse of anxiety-, depressive-, and substance use disorders. Furthermore, improvements in the quality of the review process have been observed, as demonstrated by [19], who used AL to correct noisy labels when reconstructing a systematic review dataset used in [20], who systematically reviewed the literature on the treatment of Borderline Personality Disorder. Or, consider [4], who propose to use AL to find relevant studies excluded by screeners due to screening fatigue.

Most simulation studies investigating the performance gain of AL have focussed on the work saved compared to random reading, as shown in the systematic review of simulation studies by [28]. On real-world data, Harmsen et al. [15] examined how the type of literature screening within the context of medical guideline development can affect the performance of AL-aided screening tools. They relied on clinicians’ and research methodologists' title/abstract labeling decisions and ran a simulation study. They found that the performance of AL was better for inclusions based on research methodologists than clinicians, and full-text inclusions were better for both groups. Furthermore, the abstract's quality and coherence affected the time it took to find a paper. That is, if the model classifies and ranks the records as expected, the relevant records are typically identified early in the AL-aided screening process. But, suppose one screens a dataset, and at some point, 1000 records have been screened, and 4000 records are still unseen. At this point, the screener must decide whether or not to stop screening. Let us assume that the screener uses a (naïve) stopping rule of 100 irrelevant papers in a row (more on stopping rules: [4, 8, 14, 30, 33] and the screener indeed is presented 100 irrelevant records in a row and decides to stop screening. But what if unknown to the screener, there is still a relevant record hidden in the pool of unseen records, a paper ranked much lower by the classifier? This paper is, what is called a hard-to-find because the classifier struggled to predict whether this paper is relevant. This is the reason why [27, 28] suggest switching to a different model during the screening process: to allow the model to re-rank the unseen papers using a different model to take, for example, context into account.

It is of great importance to investigate why AL models have difficulties finding some hard-to-find papers. Failing to find a relevant paper might alter the findings of a systematic review. For example, while undergoing a systematic review of the research that has been conducted on the efficacy of a treatment to inform medical guidelines, missing a relevant paper could cause a side-effect to be overlooked. Indeed, studies have demonstrated that systematic reviews that miss certain papers can alter the findings that are derived from a meta-analysis [31]. Luckily, this is not always the case, as Teijema, Hofstee et al. [27] found that removing the last-to-find relevant papers did not affect the conclusions of an original meta-analysis. They also correlated the rank-order of records across different AL models and found that ranks were more similar across classifiers than feature extractors, suggesting that the feature extractor has an influence on the rank-order of records. However, many users of AL tend to use the default settings of software concerning the model choice, and it would be unwanted if the choice of the AL model or selection of the prior knowledge influences the difficulty of finding hard-to-find relevant papers.

In this context, the time to discovery (TD) was recently proposed, enabling model performance assessment during simulation studies mimicking the AL-aided screening process using a labeled dataset [11]. The inclusion labels are treated as if these were labeling decisions from a real user, and the TD of a record measures how many records need to be screened to find a relevant paper. When multiple simulations are run on a dataset with different model specifications, the average record TD can be computed, which is the average of the TD values for a given record across simulations—also known as the average simulation TD. Examining the variance of the TD values around the average record TD allows for investigating the variability of how long it takes to find a record across different simulation set-ups (e.g., utilizing different AL models). Although the TD has been used in simulation studies for a metric of overall performance in simulation studies [27] and real-world applications [15], thus far, it has not been used to examine the variability of hard-to-find papers.

The key objective of this exploratory simulation study is to investigate how AL model selection and choice of prior knowledge affect the discovery of hard-to-find relevant papers. In the subsequent sections, the design of the simulation study will be presented, followed by an analysis and discussion. The data, scripts, and output are available on the GitHub repository for the project [7].

## Method

### Simulation set-up

To evaluate the influence of the selection of models on the variability of the time to discovery (TD) values and the stability of their rank-orders, a simulation study was run using ASReview v1.2 [2]. The Makita (v0.6.3,Teijema, Van de Schoot, et al. [34]) template generator was used to create the scripts needed to execute the two simulation studies.

We used the multiple models’ template for the first simulation study, which generates scripts to run a simulation for each classifier-feature extractor combination. We compared four classifiers (logistic regression (LR), naïve Bayes (NB), random forest (RF), support vector machine [SVM]) and three feature extractors (i.e., TF-IDF, Doc2Vec, sentence BERT [SBERT]). Note that it is not possible to combine NB with Doc2Vec or SBERT, as Doc2Vec and SBERT both produce a feature matrix that contains negative values [16, 22], while the NB classifier can only work with feature matrices containing positive values. The balancing strategy was set as dynamic resampling (double), and the query strategy was set as maximum. The prior knowledge consisted of one randomly chosen relevant and one irrelevant paper held constant across simulation runs.

For the second simulation study, to evaluate the influence of prior knowledge, we used the all relevant, fixed irrelevant (ARFI) template, which generated a script that, when run, resulted in as many simulations as there are relevant records in the datasets. Each relevant record was set as prior knowledge per run, so the number of simulations completed corresponded to the number of relevant records. Ten irrelevant records were chosen randomly and were fixed as prior knowledge across each simulation. The ARFI template was run using the default model settings: NB for the classifier, TF-IDF for the feature extractor, maximum for the query strategy, and dynamic resampling (double) for the balancing strategy.

### Data

The data was taken from the SYNERGY dataset, a free and open-source dataset on study selection for systematic reviews [10]. Specifically, we used the Radjenović dataset, collected during the screening process for a systematic review of metrics used in software fault prediction models [21]. This dataset contained 5935 records with meta-data initially screened for relevance by Radjenović and colleagues, with 48 records (8%) labeled as relevant. This dataset was chosen as it contained a small number of relevant records, which allowed for better visualization of the variability of the TD values. There were 14 duplicate records removed from the data using ASReview Datatools [1]. Additionally, one record with a missing abstract was deleted. This resulted in 5920 records in the processed data.

### Statistical analysis

The hard-to-find relevant papers were specified as the five lowest-ranked relevant records according to their average-record-TD value across the different simulation runs (i.e., those with the five highest average-record-TD values [12]. Furthermore, the average-record-TD was calculated by taking the mean of the TD values for a given record. In order to assess the variability of the TD values of the hard-to-find relevant papers across simulations, the standard deviation was calculated by squaring the deviations of the TD values from the average-record-TD, and then taking the square root of the average of these values for each record. A table of the rank-orders of the TD values across AL models was also generated (for both tables, please refer to the project’s GitHub repository; [7]).

A parallel-coordinates plot was generated to visualize the TD values and the rank order of the TD values across AL models containing the TD values of all 48 relevant papers across simulations, with each line representing the TD value for a paper across active learning models (grouped by feature extractor). A Friedman test was carried out to test for differences in TD values across models. This test was chosen as the records being compared were the same across conditions, and the TD values were not normally distributed, indicated by significant Shapiro–Wilk tests (*p* < 0.001). Post-hoc analysis was conducted between the AL models using Wilcoxon signed-rank tests, with a Bonferroni correction to account for multiple.

Furthermore, to examine the relationship between the hard-to-find relevant papers and the variability of the TD values across AL models, a Spearman’s rank correlation coefficient was computed between the average-record-TD values and their SDs across all records (this was chosen as the average-record-TDs and the SDs were not normally distributed). All scripts are publicly available and can be found in the GitHub repository for the project [7].

## Results

### Time to discovery (TD) across AL models

**Fig. 1:**
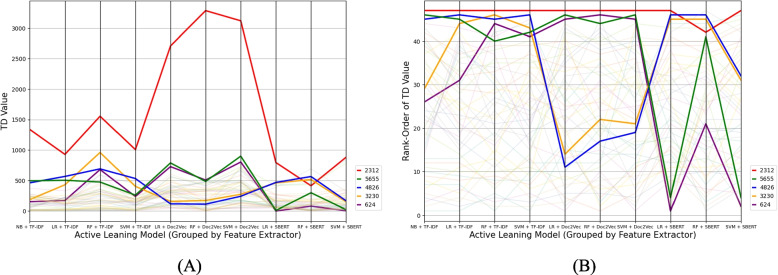
The time to discovery (TD) values (**A**) and the rank-orders (**B**) of all 48 relevant papers across 10 simulations. Each line represents the TD value for a paper across active learning models (grouped by feature extractor). The five hard-to-find relevant papers are highlighted (record IDs contained in the legend)

The hard-to-find relevant papers on average across similation runs were records number 2312 (average record TD = 1682.44; SD across simulation runs = 1075.64), 5655 (ATD = 466.67; SD = 267.56), 4826 (ATD = 415.33; SD = 209.59), 3230 (393.67; SD = 251.29), and 624 (ATD = 371.67; SD = 307.10). The digital object identifiers (DOIs), titles, and abstracts for these records can be found in the project's GitHub repository [7]. As can be seen in the parallel-coordinates plot, see Fig. 1, the TD values of these hard-to-find relevant papers are higher than the rest of the relevant papers across most model combinations. Record 2312 (in red) consistently has the highest TD value, especially when Doc2Vec was used as the feature extractor. The table containing all the TD values and the TD values’ rank-orders of the relevant records can be found in the project's GitHub repository [7].

There was a significant difference between AL models on the rank-orders of the TD values, *χ*^*2*^(9) = 139.08, *p* < 0.001, *W* = 0.33. The Bonferroni corrected p-values, test statistics, and effect sizes of the pairwise comparisons across AL models can be found in Table 1. A significant difference was found between the feature extractors (of the AL models) on the rank-orders of the TD values, *χ*^*2*^(2) = 35.62, *p* < 0.001, *W* = 0.38. Pairwise comparisons indicated that there was a significant difference between TF-IDF and Doc2Vec (*W* = 171, *p* < 0.001, *ĝ* = 0.44) and SBERT and Doc2Vec (*W* = 133, *p* < 0.001, *ĝ* = 0.55) but not between TF-IDF and SBERT (*W* = 383, *p* = 0.056, *ĝ* =  − 0.18).

**Table 1:** Pairwise-comparisons between active learning models (Bonferroni-corrected *P* values, test statistics, and effect sizes)

Model A	Model B	*p*-corr	*W*	Effect size
LR + Doc2Vec	NB + TF-IDF	< .001	76.5	0.53
NB + TF-IDF	SVM + Doc2Vec	< .001	79	− 0.63
LR + TF-IDF	RF + TF-IDF	< .001	105.5	− 0.48
SVM + Doc2Vec	SVM + TF-IDF	< .001	111	0.65
RF + SBERT	SVM + Doc2Vec	< .001	114	− 0.73
LR + TF-IDF	SVM + Doc2Vec	< .001	118	− 0.68
SVM + Doc2Vec	SVM + SBERT	< .001	76	0.63
LR + SBERT	SVM + Doc2Vec	< .001	126	− 0.68
LR + Doc2Vec	LR + TF-IDF	< .001	129	0.58
RF + TF-IDF	SVM + TF-IDF	< .001	137	0.44
RF + Doc2Vec	SVM + Doc2Vec	< .001	140.5	− 0.23
LR + Doc2Vec	SVM + TF-IDF	< .001	144	0.55
LR + Doc2Vec	RF + SBERT	< .001	145	0.64
LR + Doc2Vec	LR + SBERT	< .001	146.5	0.59
LR + Doc2Vec	SVM + SBERT	< .001	156.5	0.53
NB + TF-IDF	RF + TF-IDF	< .001	157.5	− 0.41
RF + SBERT	RF + TF-IDF	0.001	152	− 0.55
NB + TF-IDF	RF + Doc2Vec	0.002	195.5	− 0.32
RF + Doc2Vec	RF + SBERT	0.002	198	0.40
LR + Doc2Vec	SVM + Doc2Vec	0.038	257	− 0.13
RF + Doc2Vec	SVM + TF-IDF	0.038	256.5	0.33
LR + TF-IDF	RF + Doc2Vec	0.052	264.5	− 0.36
LR + SBERT	RF + TF-IDF	0.066	270.5	− 0.48
LR + Doc2Vec	RF + Doc2Vec	0.074	273	0.11
RF + TF-IDF	SVM + Doc2Vec	0.229	303	− 0.32
RF + TF-IDF	SVM + SBERT	0.292	309.5	0.41
LR + SBERT	RF + Doc2Vec	0.465	324.5	− 0.35
LR + Doc2Vec	RF + TF-IDF	0.528	327.5	0.18
RF + SBERT	SVM + TF-IDF	0.810	323.5	− 0.13
RF + SBERT	SVM + SBERT	1	346.5	− 0.14
RF + Doc2Vec	SVM + SBERT	1	355.5	0.31
LR + TF-IDF	SVM + SBERT	1	514	− 0.06
NB + TF-IDF	SVM + TF-IDF	1	515.5	0.001
NB + TF-IDF	SVM + SBERT	1	543	− 0.01
NB + TF-IDF	RF + SBERT	1	381.5	0.12
LR + TF-IDF	SVM + TF-IDF	1	355.5	− 0.04
LR + TF-IDF	RF + SBERT	1	424.5	0.08
LR + TF-IDF	NB + TF-IDF	1	480.5	− 0.04
LR + SBERT	SVM + TF-IDF	1	492	− 0.03
LR + SBERT	SVM + SBERT	1	527	− 0.05
LR + SBERT	RF + SBERT	1	383	0.10
LR + SBERT	NB + TF-IDF	1	484	− 0.03
LR + SBERT	LR + TF-IDF	1	562	0.01
RF + Doc2Vec	RF + TF-IDF	1	560	0.03
SVM + SBERT	SVM + TF-IDF	1	554	0.02

**Fig. 2:**
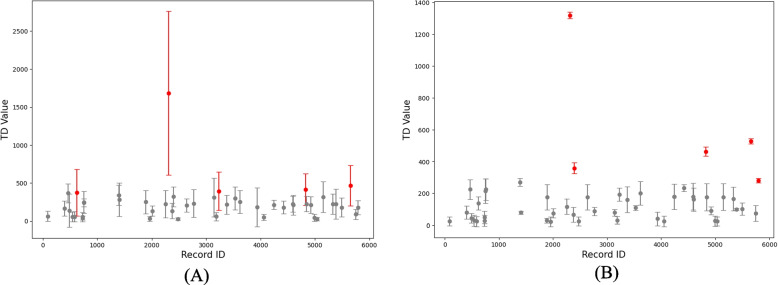
Standard deviations (SDs) of the average-record-time to discovery (TD) values for each relevant paper across active learning models (**A**) and prior knowledge (**B**). The five hard-to-find relevant papers are highlighted. Each dot represents the average-record-TD value of a paper, and the line corresponds to this value’s SD

The rank-orders of the TD values significantly differed between the classifiers (of the AL models), *χ*^*2*^(2) = 24.55, *p* < 0.001, *W* = 0.17. Pairwise comparisons indicated that there were significant differences between NB and LR (*W* = 220, *p* = 0.001, *ĝ* = 0.24), NB and RF (*W* = 192.5, *p* < 0.001, *ĝ* =  − 0.28), and NB and SVM (*W* = 146, *p* < 0.001, *ĝ* =  − 0.34). However, there were no significant differences between Log and RF (*W* = 487, *p* = 0.1, *ĝ* =  − 0.07), RF and SVM (*W* = 489, *p* = 1, *ĝ* =  − 0.04), and Log and SVM (*W* = 385.5, *p* = 0.35, *ĝ* =  − 0.011). Likewise, Bonferroni corrected p-values were used to account for multiple comparisons. A significant positive correlation was also found between the average-record-TD values and their SDs across AL models, *r*_*s*_(45) = 0.82, *p* < 0.001. The SD of the TD of the hardest-to-find paper is larger, record 2312, (*M* = 1682.44, SD = 1075.64) in comparison to that of the easiest-to-find record (i.e., the smallest average-record-TD across AL models), record 2475 (*M* = 25.78, SD = 18.36). The means and SDs of the average-record-TD values of the relevant records across AL models can be seen in Fig. 2A.

### TD across prior knowledge

The average-record-TD values and SDs of the five hardest-to-find relevant papers across different sets of prior knowledge (using NB + TF-IDF as the AL model) were records 2312 (ATD = 1318.44; SD = 20.04), 5655 (ATD = 527.56; SD = 17.65), 4826 (ATD = 462.44; SD = 29.07), 2398 (ATD 358.00; SD = 33.53), and 5791 (ATD = 279.67; SD = 13.80). Interestingly, the three hardest-to-find relevant records here are the same as across AL models. The digital object identifiers (DOIs), titles, and abstracts as well as all the TD values can be found in the project's GitHub repository [7].

**Fig. 3:**
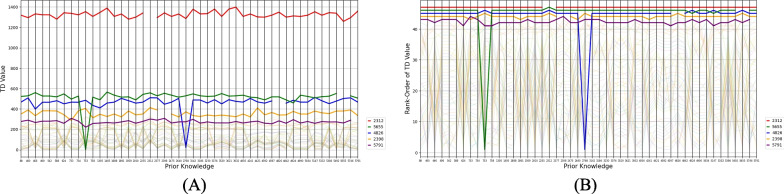
The time to discovery (TD) values (**A**) and the rank-orders of the TD values (**B**) of all 48 relevant papers across 48 simulations. Each line represents the TD values of a paper across prior knowledge. The gaps in the lines represent the condition in which a paper is used as prior knowledge. The five hard-to-find relevant papers are highlighted (record IDs listed in the legend)

As seen in Fig. 3, the TD values of each record across different prior knowledge appear rather stable across different starting papers as prior knowledge. Again, the hardest-to-find relevant paper, 2312, is consistently higher than the other records in relation to its TD value. Interestingly, for some of the simulations, the TD values of the hard-to-find papers are suddenly much lower. There was no significant difference between prior knowledge on the rank-orders of the TD values, *H*(47) = 35.863, *p* = 0.859, *W* = 0.004, nor was there a significant correlation between the average-record-TD values and their SD across different prior knowledge, *r*_*s*_(45) = 0.19, *p* = 0.187; see also Fig. 2B.

## Discussion

The present study examined the influence of the choice of active learning (AL) model and the selection of prior knowledge on the time to discovery (TD) of hard-to-find relevant papers in the context of AL-aided systematic reviewing. Notably, the hardest-to-find paper, on average across models, consistently had the highest Time to Discovery (TD) value, except for one AL model (RF + SVM). In comparison, the ranking of the remaining hard-to-find relevant papers varied considerably more across models. This observation was substantiated by the relationship found between the average-record-TDs of relevant papers and their standard deviation (SD values across multiple simulation runs with varying models or prior knowledge), demonstrating that the harder it is to find a paper, the more likely it is to vary across models.

Interestingly, the feature extractor had a more significant impact than the classifier in influencing the TD values of the hard-to-find relevant papers. This result aligns with the finding that the rank-orders of records are less correlated between feature extractors than classifiers [27]. They found that switching models after a stopping criterion (for example, halting reviewing after 50 consecutive irrelevant records) improved performance, especially when the feature extractor was changed, fitting with our findings. Moreover, Subasi [26] has argued that the feature extractor is more important than the classifier, as classification performance can be reduced if the features that are used as input for the classifier are poorly selected. This argument highlights possible reasoning for the observed discrepancy between the influence of the feature extractor and classifier on the TD values.

The TD values of the hard-to-find relevant papers on average across the default AL model in ASReview (NB + TF-IDF) did not vary with prior knowledge. This lack of variability was demonstrated through the stability of the rank-orders of the TD values of the hard-to-find relevant papers. Therefore, this further iterates that prior knowledge choice does not significantly influence how long it takes to find relevant papers while using an AL-aided screening tool.

A limitation of the present study was that we did not investigate *why* certain relevant papers are “hard-to-find” as Harmsen et al. [15] did. There may be various reasons why relevant papers are treated as less relevant by the AL model. For instance, maybe such records are incorrectly classified as relevant by the screener, or possibly they are relevant, but their titles/abstracts are dissimilar to the majority of the other relevant papers (i.e., tapping into a different cluster in the data). Furthermore, only one dataset was used to assess the variability of the TD values and the stability of their rank-orders across AL models and different prior knowledge. The limited number of datasets used in the present study may restrict the generalizability of the findings. For instance, the particular dataset chosen for the simulation study may have, by chance, contained a particular record that was consistently ranked low (i.e., ranked as less relevant) by the AL models. Therefore, it is necessary to study the influence of AL model choice on the TD values of the hard-to-find relevant papers across multiple datasets to determine whether the findings from the present study are generalizable. For instance, utilizing the complete SYNERGY dataset would enhance the robustness of the aforementioned conclusions [10].

Future research should examine the characteristics of the hard-to-find relevant papers (e.g., the content of their titles and abstracts) to provide insights into why such papers have high TD values. For example, Harmsen et al. [15] demonstrated that the group that conducted the systematic review affected the efficiency of AL-aided screening. For example, clinicians labeled some papers as relevant, that were identified only very late in the process, and which research methodologists did not even include. The researchers initiated focus groups to discuss the hard-to-find papers to make sure these were correctly labeled as relevant or irrelevant, and they tried to identify reasons for differences. For example, the mismatch in labels occurred due to the inclusion criteria being slightly differently interpreted between particular groups of individuals, i.e., clinicians versus research methodologists.

The present study has important implications for the field of AL-aided screening tools as it highlights the use of the TD metric to help locate and assess the variability of the hard-to-find relevant papers across different simulation set-ups. Previous research on TD has examined the ATD (or average-simulation TD in the context of simulation studies) across different models and datasets [12]. In contrast, our study was the first to use the average-record-TD to locate the hard-to-find relevant papers across different models and prior knowledge. Furthermore, the study’s findings emphasize the influence of AL model selection, specifically the feature extractor, on the difficulty of discovering hard-to-find relevant records in a dataset. Therefore, the field can build off the current findings with future research to find an optimal model for decreasing the chances of hard-to-find relevant papers being ranked less relevant by the screening tool.

As was found, the choice of AL model significantly affects the time it takes to locate hard-to-find relevant papers within a dataset, and this further demonstrates the importance of model selection prior to the screening process. Importantly, the current findings substantiate the recommendation proposed by Teijema, Hofstee et al. [27] to switch models after a stopping criterion. Choosing a different model after such a threshold, in particular, another feature extractor may decrease the TD values of the hard-to-find relevant papers. However, this recommendation depends on why the hard-to-find relevant papers are difficult to find. For example, switching models is not advised if a hard-to-find relevant paper was incorrectly classified, while this is suggested for hard-to-find papers that are dissimilar to the other relevant papers in terms of their content yet are still relevant.

### Data statement

The data that was used in the present study did not contain any sensitive information about persons. The data consisted solely of titles, abstracts, digital object identifiers (DOIs), and inclusion labels from the screening process for a systematic review of software fault prediction metrics from the field of computer science [21]. The data is open-source and can be accessed via the SYNGERGY dataset [10].
